# An adult case of junctional tachycardia diagnosed by premature atrial contractions and atrial overdrive pacing

**DOI:** 10.1002/joa3.12841

**Published:** 2023-03-10

**Authors:** Hironori Nakamura, Hidehira Fukaya, Naruya Ishizue, Jun Kishihara, Junya Ako

**Affiliations:** ^1^ Department of Cardiovascular Medicine Kitasato University School of Medicine Sagamihara Japan

**Keywords:** atrial overdrive pacing, junctional tachycardia, premature atrial contraction

## Abstract

We report an adult case with JT, which could be differentiated from slow‐fast AVNRT by premature atrial contractions and atrial overdrive pacing.

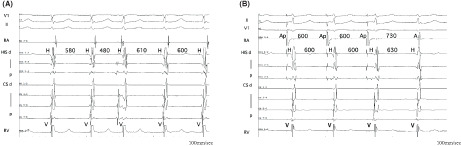

A 62‐year‐old man with a history of hypothyroidism presented to our hospital with recurrent palpitations. A 12‐lead electrocardiogram (ECG) documented a regular narrow QRS complex tachycardia (NCT) (Figure 1). The ECG during normal sinus rhythm exhibited no ventricular preexcitation. Echocardiography showed no findings to suggest underlying cardiac disease. An electrophysiological study (EPS) was performed after obtaining informed consent.

**FIGURE 1:**
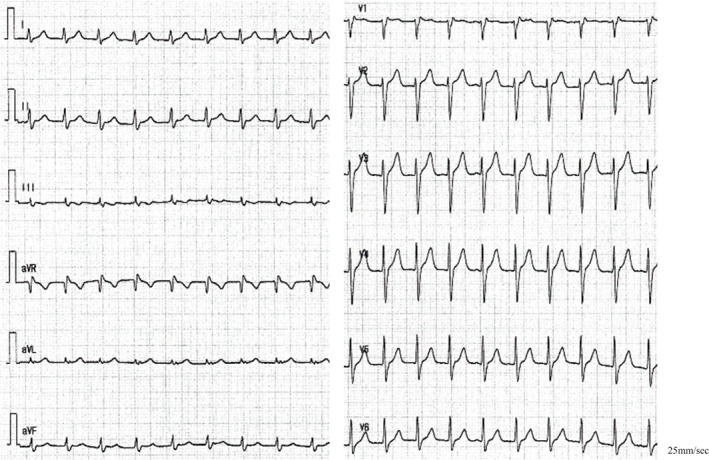
The 12‐lead ECG shows a regular narrow QRS complex tachycardia.

An EPS was performed in a nonsedated state. The A–H and H–V intervals were 94 and 35 ms during sinus rhythm with a sinus cycle length of 1150 ms. Right ventricular (RV) pacing revealed that the retrograde activation was concentric, and the earliest atrial activation was recorded in the His bundle (HB) region. A clinical NCT was induced with atrial extra or burst stimulation without a jump‐up of the AH interval (Figure 2A). Furthermore, a positive correlation between the S1–S2 interval and tachycardia return cycle was observed during tachycardia induction by atrial extrastimuli (Figure 2B,C), which suggested that the mechanism of this tachycardia is not reentry. The tachycardia cycle was slightly fluctuating and accelerated after the isoproterenol was administered. The earliest atrial activation site during tachycardia was recorded in the HB region. During right ventricular overdrive pacing (RVOP), an atrial activation sequence was identical to the NCT. The difference between the post‐pacing interval and tachycardia cycle length corrected by an atrioventricular nodal conduction delay was 136 ms (>110 ms) with a V‐A‐V response after RVOP, which was less likely to be an atrioventricular reentry tachycardia or atrial tachycardia.^3^, ^4^ A spontaneous early PAC advanced the His potential immediately, and the tachycardia was still sustained (Figure 3A). Atrial overdrive pacing (AOP) from a high right atrial site resulted in an A‐H‐H‐A response (Figure 3B). Three‐dimensional electroanatomic mapping during tachycardia showed that the earliest atrial activation site was located near the anterior HB region. Finally, we diagnosed the NCT as junctional tachycardia (JT) and did not perform catheter ablation, considering the risk of atrioventricular block.

**FIGURE 2:**
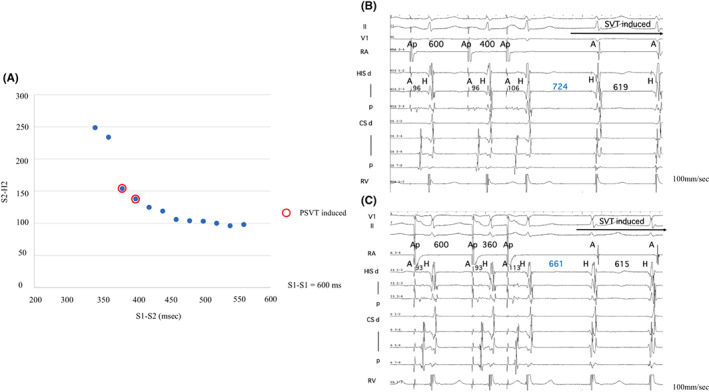
(A) The atrioventricular conduction curve during programmed atrial stimulation is shown. The SVT was induced by an atrial extrastimulus pacing (red circle) without a jump‐up phenomenon. (B) After the last atrial‐paced beat (S1–S1 = 600 ms, S1–S2 = 400 ms), the return cycle of the tachycardia was 724 ms. (C) After the last atrial‐paced beat (S1–S1 = 600 ms, S1–S2 = 360 ms), the return cycle of the tachycardia was 661 ms. An inverse correlation of the return cycle was not observed. A: atrial potential, Ap: atrial pacing, CS: coronary sinus; d: distal, H: His potential, HIS: a bundle of His, p: proximal, RA: right atrium, RV: right ventricle, SVT: supraventricular tachycardia.

**FIGURE 3:**
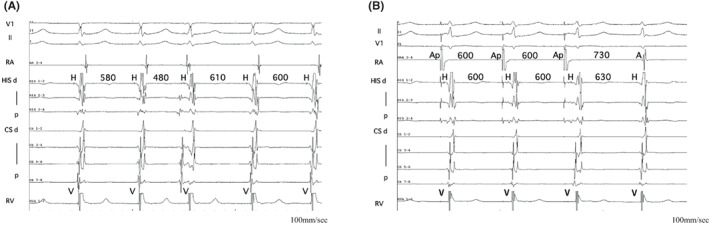
(A) A spontaneous PAC, which occurred earlier than the His refractory period, advanced the His potential without tachycardia termination. (B) trial overdrive pacing from a high right atrial site exhibited an A‐H‐H‐A response. A: atrial potential, Ap: atrial pacing, CS: coronary sinus, d: distal, H: His potential, HIS: a bundle of His, p: proximal, RA: right atrium, RV: right ventricle, SVT: supraventricular tachycardia.

The mechanism of JT is abnormal automaticity or triggered activity.^1^ In the present case, tachycardia was induced by programmed atrial stimulation. Of note, the return cycle length of the tachycardia did not inversely correlate with the S1–S2, suggesting that the mechanism of this tachycardia was not reentry and also ruled out a double ventricular response. Further, atrial scan pacing or spontaneous PACs during the tachycardia can differentiate slow‐fast atrioventricular nodal reentrant tachycardia (S/F AVNRT) and JT. When a PAC at a timing when the His is refractory advances the tachycardia, the slow pathway is within the circuit, which supports the diagnosis of S/F AVNRT. When a PAC earlier than the His refractory period immediately advances the His potential without termination of the tachycardia, the retrograde fast pathway is not included in the circuit, that is, it is not likely S/F AVNRT, resulting in the diagnosis of JT.^2^

JT and AVNRT can be differentiated by assessing the resumed tachycardia after the AOP. AOP during JT transiently suppresses tachycardia. After cessation of the AOP, JT restarts with a junctional beat led by a His signal, an A‐H‐H‐A response.^3^, ^4^ On the other hand, the last atrial‐paced beat during S/F AVNRT conducts anterogradely down the slow pathway and propagates through the His–Purkinje system while echoing up the fast pathway, resulting in an A‐H‐A response. It should be noted that AVNRT may be misdiagnosed as JT in rare cases when a double ventricular response is induced. A comprehensive evaluation, combining the response of the PAC during the tachycardia and the return cycle of the tachycardia after programmed stimulation from the atria, would help the diagnosis.

## CONFLICT OF INTEREST STATEMENT

The authors declare that there is no conflict of interest.

## ETHICS STATEMENT

The research related to human use has complied with all the relevant national regulations and institutional policies and is in accordance with the tenets of the Helsinki Declaration and has been approved by the author's institutional review board or equivalent committee.

## PATIENT CONSENT STATEMENT

Informed consent has been obtained from all individuals included in this study.

## CLINICAL TRIAL REGISTRATION

None.

## Data Availability

The data sets generated and/or analyzed during the current study are available from the corresponding author upon reasonable request.
